# The ground has shifted under PEPFAR: what does that mean for its future?

**DOI:** 10.1002/jia2.26396

**Published:** 2024-11-22

**Authors:** Jennifer Kates, Brian Honermann, Gregorio Millett

**Affiliations:** ^1^ Global Health & HIV Policy, KFF Washington DC USA; ^2^ Public Policy, amfAR Washington DC USA

1

PEPFAR, the U.S. global HIV programme, has been credited with saving 25 million lives and changing the trajectory of the HIV/AIDS pandemic [1]. Last year, more than 20 million people were on antiretroviral therapy with support from PEPFAR, almost 2 million were newly enrolled on pre‐exposure prophylaxis and 327,000 healthcare workers were directly supported by the program. PEPFAR also estimates that more than 5 million babies have been born without HIV. In addition, studies have found that PEPFAR funding is significantly associated with several, positive, knock‐on effects beyond HIV, including increases in the gross domestic product (GDP) per capita growth rate, educational retention and childhood vaccination rates [2].

Created in 2003 in the United States by a Republican President, with strong, bipartisan support in Congress at the time, PEPFAR has largely maintained that support across multiple administrations and congresses, often standing outside the political fray in Washington, DC. But the ground upon which PEPFAR sits has shifted in fundamental ways, perhaps most obviously manifest in the challenges it recently faced in securing a 5‐year reauthorization [3]. These shifts are multifaceted and intertwined and, in most cases, not specific to PEPFAR or HIV, but taken together, suggest a “rethink” for PEPFAR's next phase. Here, we explore some of these shifts and the questions they pose going forward, questions that have become even more important given the outcome of the U.S. election; a second Trump administration and a changing balance in Congress likely mean, at a minimum, even greater scrutiny of the programme.

One of the greatest shifts is in the global economy. While recovering, it continues to experience the economic effects of the COVID‐19 pandemic, with GDP growth remaining below historic averages. Fiscal space is further strained by high inflation and the ongoing costs of multiple wars and humanitarian assistance [4]. For donor governments, these fiscal strains present challenges for financing health and development needs, including for HIV, and many are shifting away [5]. For low‐ and middle‐income countries, rising debt burden threatens their economic recovery, with many poorer now than before COVID‐19 [4].

More broadly, reports have found that the human rights environment in many countries is deteriorating, with negative effects on health [6]. This has particular implications for HIV given that many of the populations most affected—men who have sex with men, transgender women, people who use drugs and other marginalized groups—already face human rights barriers that put them at increased risk for HIV and complicate the ability to control HIV [6]. There is also evidence that civic space is closing in many localities, making it more difficult for civil society organizations to operate and organize and presenting new challenges for HIV and other health needs [7].

Other shifts include the rise in political polarization, mistrust and misinformation. For example, trust in science and health institutions has declined, and is increasingly diverging along partisan lines [8]. The ability to share and spread information widely and quickly has contributed to a concomitant rise in mis‐ and dis‐information generally and for health specifically [5].

Finally, the global health and development space has become more complex and increasingly “crowded” in recent years with many overlapping, and in some cases, competing challenges. This includes a current “replenishment traffic jam,” [9] with multiple institutions calling for donor government funding from the same constrained pots at the same time. HIV—in part because of the enormous success of PEPFAR and the Global Fund to Fight AIDS, Tuberculosis and Malaria—does not evoke the same sense of urgency it once did, making its funding case more challenging. Indeed, many donor governments have reduced their spending on HIV, leaving the United States to shoulder an increasing burden of the response [10]. Even the United States, which continues to be the largest donor government to HIV in the world, reached its funding high‐water mark in 2010 [11]. But the lessons from other infectious diseases, such as malaria and tuberculosis, show that when attention and funding attenuate, these diseases can resurge [12].

These broader trends have implications for PEPFAR support and its ongoing story, exacerbated by the small and diminishing number of members of the U.S. Congress who were in office when PEPFAR was created. As a result, Congress and others are increasingly looking to PEPFAR for more concrete plans about its future [13].

Given this changing environment, there may be new questions to consider, including:

- What lessons have been learned from PEPFAR's prior experience with transitioning countries and programmes from support? For example, PEPFAR has reduced funding to several countries over time (e.g. Botswana, Rwanda, Thailand [11]); shifted funding within countries (e.g. Tanzania [14]); moved from supporting a country's full HIV programme to serving as technical assistance partner (e.g. Viet Nam [15]); and transitioned some service categories to country governments (e.g. blood safety services in 14 countries [16]).
- What are the specific plans to transition functions (service delivery, training and ultimately financing) of HIV programmes to countries over time? How will those be assessed, modified and monitored for backsliding? Will PEPFAR's “sustainability roadmaps” [17] with countries, expected later this year, contain the detail necessary for Congress and other stakeholders to fully visualize this process and the timelines involved?
- Is it time to consider strategies that have not generally been used by U.S. foreign aid programmes—but are by several other international development institutions—to more formally transition countries from PEPFAR financial support , such as progressive co‐financing requirements and income thresholds that gradually reduce eligibility for support over time?
- How can PEPFAR use its unique strengths to achieve more sustainable responses? Such strengths include: its use of data to document success and identify remaining gaps; its market‐shaping capacity, which has helped to drive down the cost of antiretroviral drugs and other commodities over time; its increased focus on key and vulnerable populations who often face discrimination and stigma including from their own governments, and its proven ability to leverage PEPFAR funding to improve health infrastructure and respond to other outbreaks and infectious disease threats.

On this World AIDS Day, these questions and others could offer new opportunities to rethink PEPFAR's future, proactively recasting and reframing it, while keeping a focus on the goal of ending the AIDS pandemic as a public health threat. This is particularly important given the outcome of the U.S. election, as well as multiple other elections in donor countries, and diminishing interest in addressing HIV.

## COMPETING INTERESTS

The authors declare no competing interests.

## AUTHORS’ CONTRIBUTIONS

JK wrote the main commentary. BH and GM contributed to the commentary and provided edits.
